# Supplementary material for: Analog–digital hybrid computing with SnS2 memtransistor for low-powered sensor fusion
Source: Nat Commun. 2022 May 19;13:2804. doi: 10.1038/s41467-022-30564-5 (PMC9119935; doi:10.1038/s41467-022-30564-5)
Supplement: Supplementary file 1 — Supplementary information [file 41467_2022_30564_MOESM1_ESM.pdf]

Supplementary information for

Analog–digital hybrid computing with SnS_2 memtransistor for low-powered sensor fusion

*Shania Rehman, Muhammad Farooq Khan, Hee-Dong Kim, and Sungho Kim**

Department of Electrical Engineering and Convergence Engineering for Intelligent Drone,

Sejong University, Seoul, Korea 05006

Corresponding author: *Sungho Kim*, email: sungho85kim@sejong.ac.kr

Supplementary Note 1. The summary of relationship among Euler angles, angular velocities, and accelerations

The gyroscope measures angular velocities projected on its sensing axes, i.e., p, q, and r. The relationship between Euler angles and angular velocities is known as

$$\begin{pmatrix} \dot{\phi} \\ \dot{\theta} \\ \dot{\psi} \end{pmatrix} = \begin{pmatrix} 1 & \sin\phi\tan\theta & \cos\phi\tan\theta \\ 0 & \cos\phi & -\sin\phi \\ 0 & \sin\phi/\cos\theta & \cos\phi/\cos\theta \end{pmatrix} \begin{pmatrix} p \\ q \\ r \end{pmatrix} \tag{S1}$$

By substituting measured angular velocities into Eq. (S1) and integrating, the rotations of the drone (Euler angles) can be obainted. However, because the measurement noise is inevitably included, Eq. (S1) is difficult to use in practice unless the the integration time is very short.

Another way to estimate Euler angles is to use an accelerometer. The accelerometer measure difference between any linear acceleration in the accelerometer's reference frame and the earth's gravitational field vector. The measured accelerations (A_x, A_y, and A_z) is determined by

$$\begin{pmatrix} A_x \\ A_y \\ A_z \end{pmatrix} = \begin{pmatrix} \dot{u} \\ \dot{v} \\ \dot{w} \end{pmatrix} + \begin{pmatrix} 0 & w & -v \\ -w & 0 & u \\ v & -u & 0 \end{pmatrix} \begin{pmatrix} p \\ q \\ r \end{pmatrix} + g \begin{pmatrix} \sin\theta \\ -\cos\theta\sin\phi \\ -\cos\theta\cos\phi \end{pmatrix} \tag{S2}$$

where u, v, and w represent the velocities of the center of gravity, and g is gravitational acceleration. Assumming that the drone is moving at a constant speed without any rotation,

$$\begin{gathered} \dot{u} = \dot{v} = \dot{w} = 0 \\ p = q = r = 0 \end{gathered} \tag{S3}$$

Then, Eq.(S2) can be simplified as

$$\begin{pmatrix} A_x \\ A_y \\ A_z \end{pmatrix} = g \begin{pmatrix} \sin\theta \\ -\cos\theta\sin\phi \\ -\cos\theta\cos\phi \end{pmatrix} \tag{S4}$$

Conseuquently, Euler angles can be estimated as

$$\phi = \tan^{-1}\left(A_y / A_z\right)$$
$$\theta = \tan^{-1}\left(A_x / \sqrt{{A_y}^2 + {A_z}^2}\right) \quad \text{(S5)}$$

Note that the estimation of yaw (ψ) from the accelerometer is not reliable; because most of the time the drone will be flying close to parallell with the axis of Earth's gravitational pull, any rotation around *z*-axis (yaw) will have very little to no effect on the accelerometer output. Therefore, a magnetometer is additionally required to accurately estimate yaw. In our study, we will focus on the estimation of roll and pitch only through sensor fusion.

Supplementary Note 2. The additional information of experimental setup

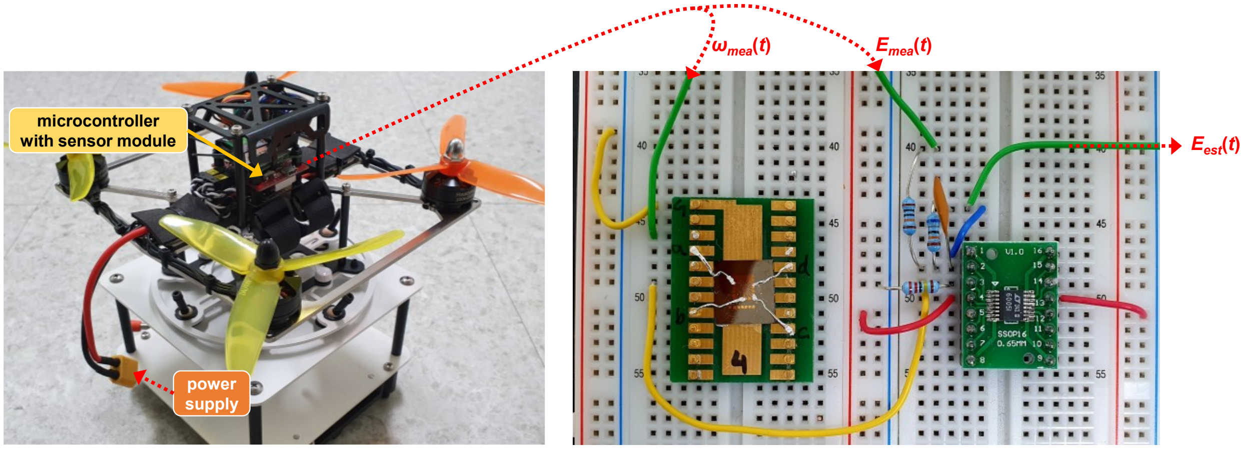

Figure S1. The images of the digital component (microcontroller & sensor module) and the analog component (memtransistor-based analog Kalman filter circuit).

2-1. The specifications of the microcontroller and the sensor module

1) microcontroller (ATmega32U4):

- 16 MIPS (Million Instroctuions Per Second) with the clock speed of 16 MHz

2) IMU sensor module (MPU6050):

- the gyroscope measures the angular velocities in the rage of ±250 °/s. The power spectral density (at 10 Hz) of the gyroscope noise is 0.005 °/s·√Hz.

- the accelerometer measures the accerlerations in the range of ±2*g*, where *g* is the acceleration of gravity. The power spectral density (at 10 Hz) of the accelerometer noise is 400 μ*g*/√Hz.

- intentionally, the built-in digital filtering function was disabled in our experiment

- the sensor module sends the data to the microcontroller through I^2C serial communication bus.

2-2. The raw data of angular velocities and the accelerations

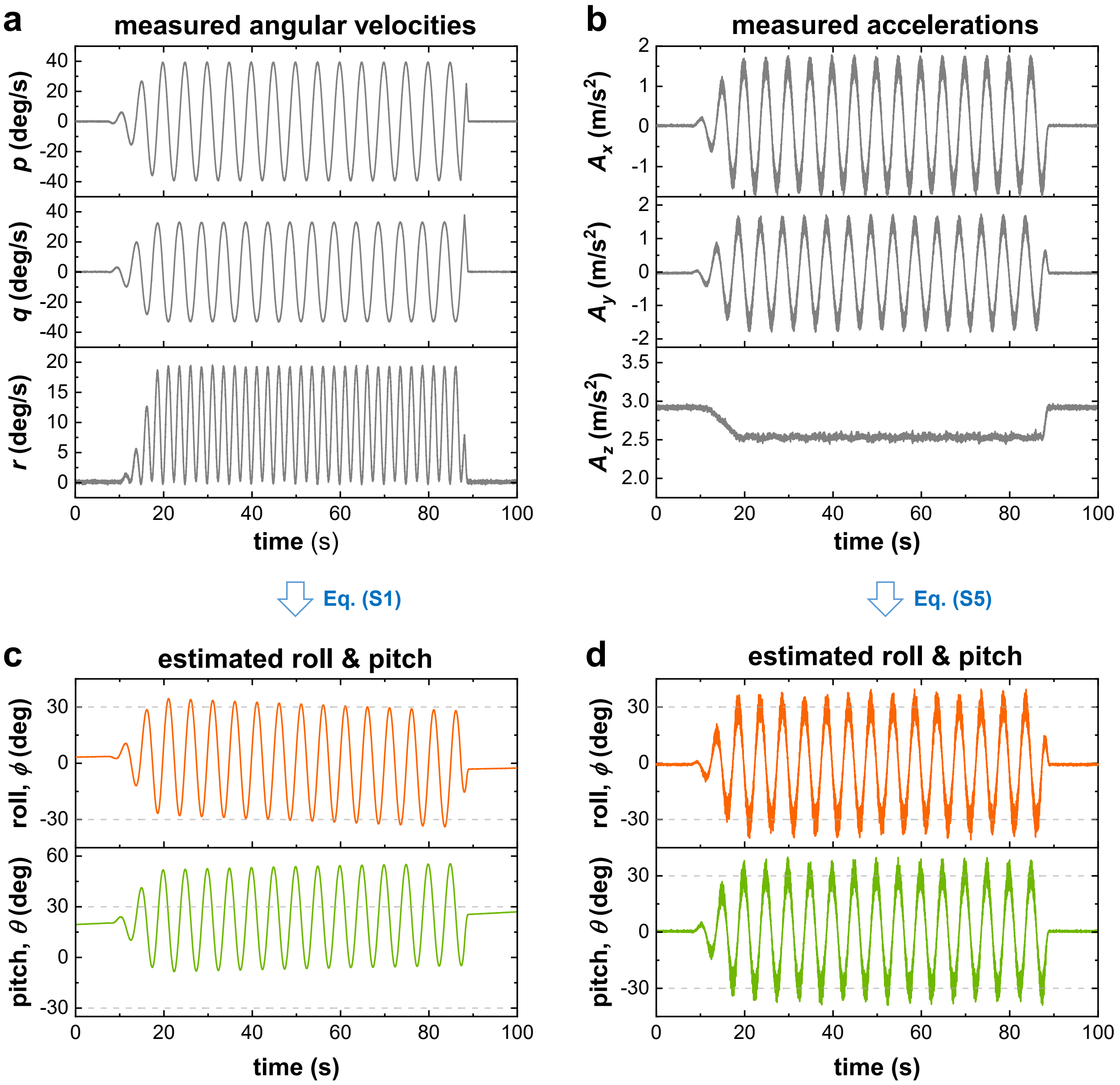

Figure S2. (a) The raw data of angular velocities. (b) The raw data of acclerations. (c) The estimated roll and pitch based on angular velocities using Eq. (S1). The dotted line represents ±30°, which is the actual oscillation amplitude of the drone. (d) The estimated roll and pitch based on acclerations using Eq. (S5).

Fig. S2a and Fig. S2b show the measured raw data of angular velocities (p, q, and r) and accelerations (A_x, A_y, and A_z), respectively. Note that the measured angular velocities do not

contain any noticeable noise but the measured accelerations contain a high frequency noise. The effect of a sensor's noise becomes more obvious after estimating the Euler angles. Using Eq. (S1) and Eq. (S5), the roll and pitch angles can be calculated (Fig. S2c and Fig. S2d). When the data of only angular velocities are used (Fig. S2c), the estimated roll and pitch angles are sufficiently consistent with the drone's actual oscillation (the amplitude of ±30°). However, a drift in which the error gradually accumulates over time is observed. Conversely, when the data of accelerations are only used (Fig. S2d), there is no drift, but the estimated roll and pitch angles include a high frequency noise. In addition, the oscillation range is also overestimated. Therefore, due to an intrinsic bias instability from the gyroscope and a high frequency noise from the accelerometer, Euler angles cannot be estimated accurately without exploiting the sensor fusion.

Supplementary Note 3. The traditional Kalman filter algorithm

3-1. Basic principle of traditional discrete-time Kalman filter algorithm (software-based Kalman filter)

Kalman filters are used to estimate states based on linear dynamic systems in state space format. The process model defines the evolution of the state from timestep $k-1$ to timestep k as:

$$x_k = Fx_{k-1} + Bu_{k-1} + w_{k-1} \tag{S6}$$

where F is the state transition matrix applied to the previous state vector x_{k-1}, B is the control-input matrix applied to the control vector u_{k-1}, and w_{k-1} is the process noise vector that is assumed to be zero-mean Gaussian with the covariance Q, i.e., $w_{k-1} \sim N(0,Q)$. The process model is paired with the measurement model that describes the relationship between the state and the measurement at the current timestep k as:

$$z_k = Hx_k + v_k \tag{S7}$$

where z_k is the measurement vector, H is the measurement matrix (or observation matrix), and v_k is the measurement noise vector that is assumed to be zero-mean Gaussian with the covariance R, i.e., $v_{k-1} \sim N(0,R)$.

The role of the Kalman filter is to provide estimate of x_k at timestep k, given the initial estimate of x_0, the series of measurement $z_1, z_2, \cdots, z_k$, and the information of the system described by F, B, H, Q, and R. Kalman filter algorithm consists of two stages: prediction and update. In the following equations, the hat operator, ^, means an estimate of a variable. That is, $\hat{x}_k$ is an estimate

of x_k. The superscripts $-$ and $+$ denote predicted (*prior*) and updated (*posterior*) estimates, respectively.

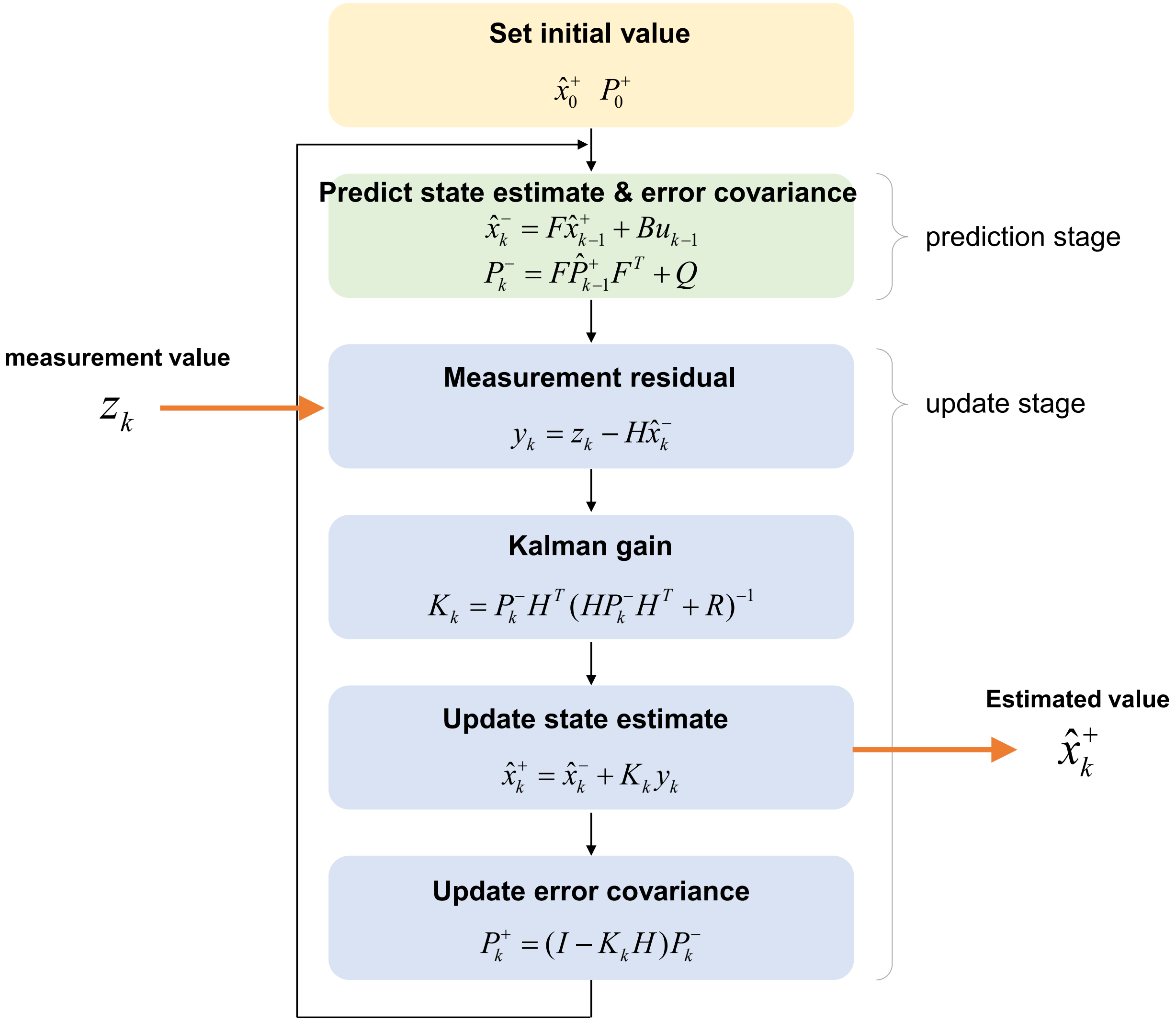

Figure S3. The flowchart of the Kalman filter algorithm.

3-2. The relationship between Euler angle and quaternion in the drone

Euler angles are simple and intuitive, and they lend themselves well to simple analysis and control. However, Euler angles are limited by a phenomenon called "Gimbal Lock", which is the

situation when the orientation cannot be uniquely represented using Euler Angles. For this reason, a quaternion formulation (q_1, q_2, q_3, q_4) to represent the rotations of the drone is more preferred,

$$q = q_1 + q_2 i + q_3 j + q_4 k, \qquad \sum_{i=1}^{4} q_i = 1 \tag{S8}$$

Transform from Euler angle to quaternion is given by

$$\begin{pmatrix} q_1 \\ q_2 \\ q_3 \\ q_4 \end{pmatrix} = \begin{pmatrix} \cos\frac{\phi}{2}\cos\frac{\theta}{2}\cos\frac{\psi}{2} + \sin\frac{\phi}{2}\sin\frac{\theta}{2}\sin\frac{\psi}{2} \\ \sin\frac{\phi}{2}\cos\frac{\theta}{2}\cos\frac{\psi}{2} - \cos\frac{\phi}{2}\sin\frac{\theta}{2}\sin\frac{\psi}{2} \\ \cos\frac{\phi}{2}\sin\frac{\theta}{2}\cos\frac{\psi}{2} + \sin\frac{\phi}{2}\cos\frac{\theta}{2}\sin\frac{\psi}{2} \\ \cos\frac{\phi}{2}\cos\frac{\theta}{2}\sin\frac{\psi}{2} - \sin\frac{\phi}{2}\sin\frac{\theta}{2}\cos\frac{\psi}{2} \end{pmatrix} \tag{S9}$$

In contrast, transform from quaternion to Euler angle is given by

$$\begin{pmatrix} \phi \\ \theta \\ \psi \end{pmatrix} = \begin{pmatrix} \tan^{-1}\dfrac{2(q_1 q_2 + q_3 q_4)}{1 - 2(q_2^{\,2} + q_3^{\,2})} \\ \sin^{-1}\left[2(q_1 q_3 - q_4 q_2)\right] \\ \tan^{-1}\dfrac{2(q_1 q_4 + q_2 q_3)}{1 - 2(q_3^{\,2} + q_4^{\,2})} \end{pmatrix} \tag{S10}$$

3-3. The sensor fusion with traditional Kalman filter algorithm

The state vector for the rotation of the drone is represented by the quaternion,

$$x = \begin{pmatrix} q_1 \\ q_2 \\ q_3 \\ q_4 \end{pmatrix} \tag{S11}$$

The relationship between the angular velocities and the quaternion is known as

$$\begin{pmatrix} \dot{q}_1 \\ \dot{q}_2 \\ \dot{q}_3 \\ \dot{q}_4 \end{pmatrix} = \frac{1}{2} \begin{pmatrix} 0 & -p & -q & -r \\ p & 0 & r & -q \\ q & -r & 0 & p \\ r & q & -p & 0 \end{pmatrix} \begin{pmatrix} q_1 \\ q_2 \\ q_3 \\ q_4 \end{pmatrix} \tag{S12}$$

In the Kalman filter algorithm, we are interested in the the transition of the state vector from a previous timestep $t - \Delta t$ to the current timestep t. For notational convenience, we represent the previous time step with $k-1$ and the current time with k. Because F in Eq. (S6) should be a discretized matrix. we need to transform Eq. (S12) as a discretized form,

$$\begin{pmatrix} \dot{q}_1 \\ \dot{q}_2 \\ \dot{q}_3 \\ \dot{q}_4 \end{pmatrix}_k = \left[I + \Delta t \cdot \frac{1}{2} \begin{pmatrix} 0 & -p & -q & -r \\ p & 0 & r & -q \\ q & -r & 0 & p \\ r & q & -p & 0 \end{pmatrix} \right] \begin{pmatrix} q_1 \\ q_2 \\ q_3 \\ q_4 \end{pmatrix}_{k-1} \tag{S13}$$

Then, the state transition matrix F in Eq. (S6) becomes

$$F = I + \Delta t \cdot \frac{1}{2} \begin{pmatrix} 0 & -p & -q & -r \\ p & 0 & r & -q \\ q & -r & 0 & p \\ r & q & -p & 0 \end{pmatrix} \tag{S14}$$

In addition, because all state variables (q_1, q_2, q_3, and q_4) should be estimated, the measurement matrix H becomes the identity matrix.

$$H = \begin{pmatrix} 1 & 0 & 0 & 0 \\ 0 & 1 & 0 & 0 \\ 0 & 0 & 1 & 0 \\ 0 & 0 & 0 & 1 \end{pmatrix} \tag{S15}$$

Finally, Fig. S3 show the the flowchart of the Kalman filter algorithm for the sensor fusion in the drone. Initial guess of the state vector $\hat{x}_0^+$ and the initial guess of the error covariance matrix P_0^+ are arbitrary chosen as

$$\hat{x}_0^+ = \begin{pmatrix} 1 \\ 0 \\ 0 \\ 0 \end{pmatrix}, \qquad P_0^+ = \begin{pmatrix} 1 & 0 & 0 & 0 \\ 0 & 1 & 0 & 0 \\ 0 & 0 & 1 & 0 \\ 0 & 0 & 0 & 1 \end{pmatrix} \tag{S16}$$

Similary, Q and R are arbitrary chosen as

$$Q = \begin{pmatrix} 0.001 & 0 & 0 & 0 \\ 0 & 0.001 & 0 & 0 \\ 0 & 0 & 0.001 & 0 \\ 0 & 0 & 0 & 0.001 \end{pmatrix}, \quad R = \begin{pmatrix} 10 & 0 & 0 & 0 \\ 0 & 10 & 0 & 0 \\ 0 & 0 & 10 & 0 \\ 0 & 0 & 0 & 10 \end{pmatrix} \tag{S17}$$

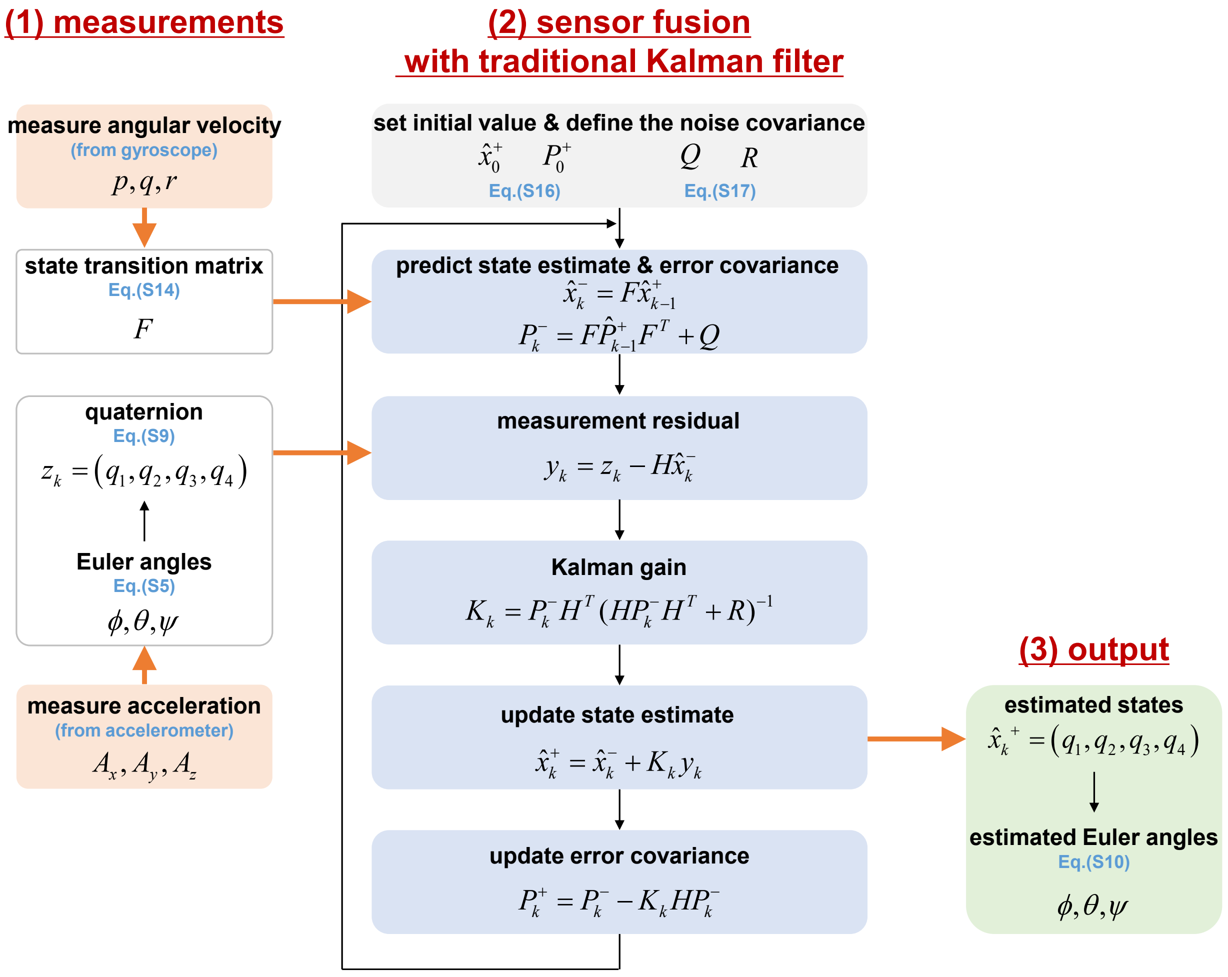

Figure S4. The flowchart of the sensor fusion procedure based on the traditional Kalman filter

Supplementary Note 4. The additional analysis of SnS_2 memtransistor

4-1. Energy dispersive X-ray (EDX) analysis

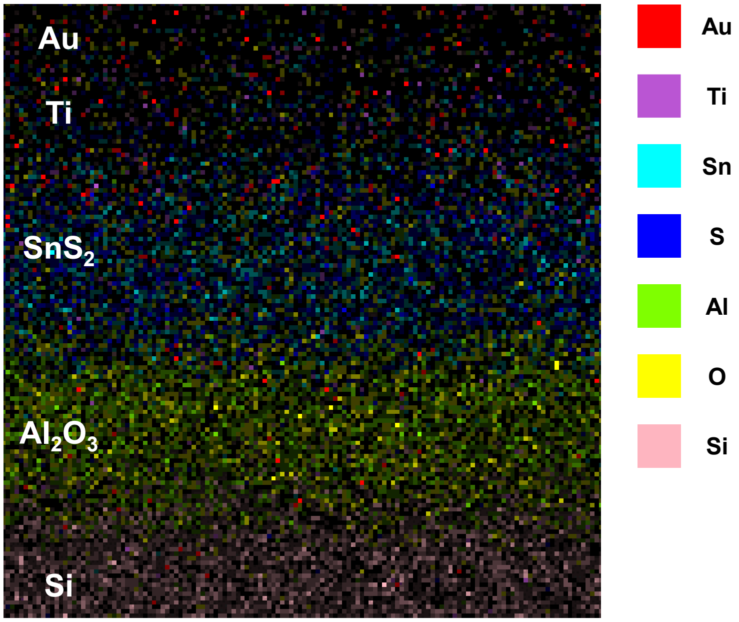

Figure S5. EDX result of the SnS_2 memtransistor.

4-2. Output characteristics

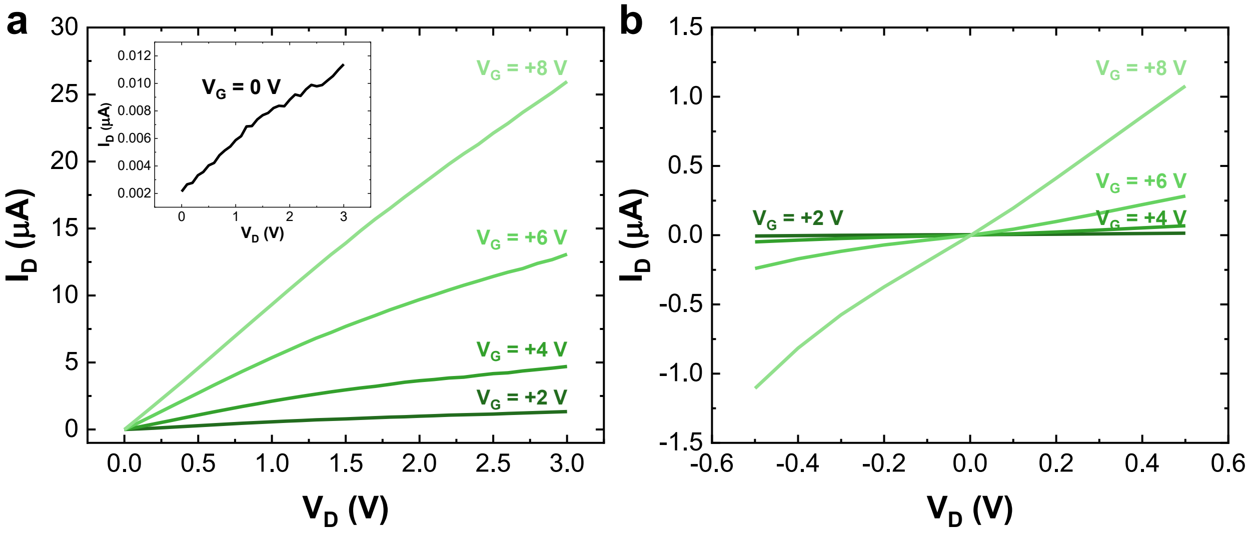

Figure S6. (a) Output characteristic of SnS_2 memristor. (b) Ohmic behaviors (linear curves near 0 V) confirms the low contact resistance between Ti/Au electrodes and SnS_2.

4-3. Update-verify feedback method

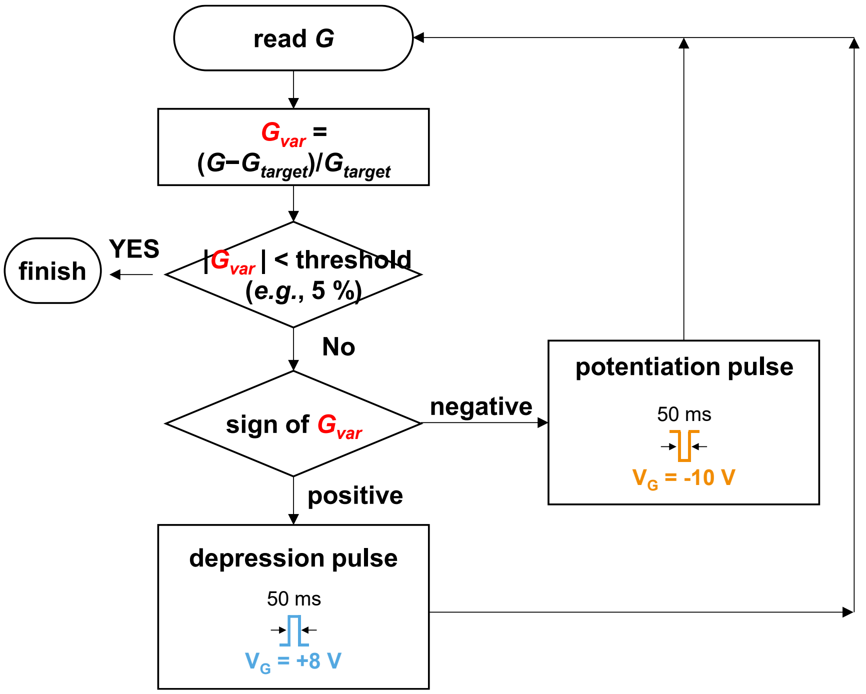

Figure S7. The flow chart for the update-verify feedback method.

Fig. S7 shows a flow chart for the update-verify process for the precise control of device conductance (*G*). The following process is repeated until the desired *G* is obtained.

(1) At the outset, G_{target} and a threshold value must be defined. G_{target} is the desired *G* value. The threshold value is the acceptable limit of the relative error between *G* and G_{target}. In our experiment, we set the threshold value as ±5%.

(2) *G* is measured using a read pulse (V_G = 0 V, V_D = 0.5 V, 50 ms) and the relative error (G_{var}) is calculated.

(3) If G_{var} is within the predefined threshold (i.e., ±5 %), the feedback process is considered complete and the process is stopped.

(4) Else, action is taken based on the sign of G_{var}. For negative G_{var}, a potentiation pulse (V_G = −10 V, V_S = V_D = 0 V, 50 ms) is applied to increase G. Meanwhile, for positive G_{var}, a depression pulse (V_G = +8 V, V_S = V_D = 0 V, 50 ms) is applied to decrease G.

In the experimental demonstration (Fig. 2e), approximately 10 repeated feedback processes are required to achieve the desired G value.

4-4. Measurement result in vacuum condition

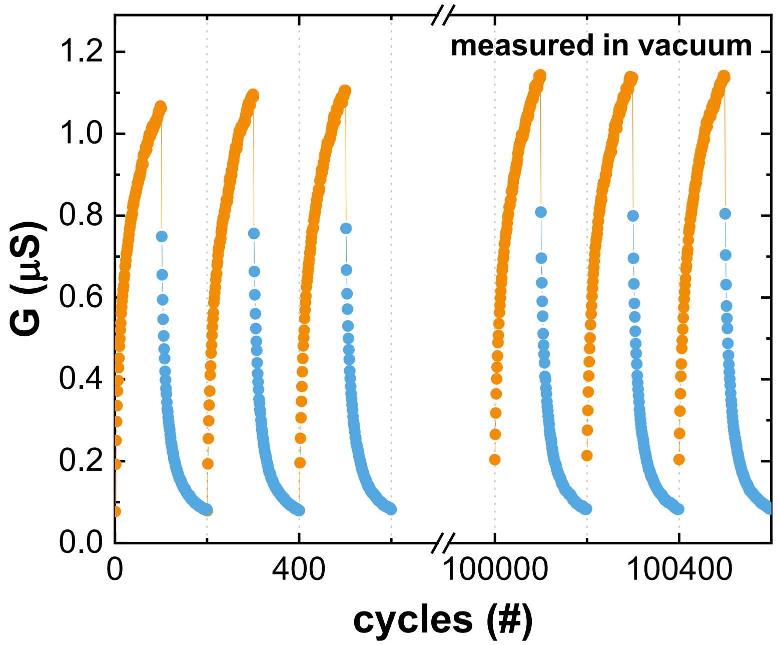

Figure S8. The resistive switching of SnS_2 memtransistor measured in a vacuum condition.

4-5. Energy band diagram of SnS_2 memtransistor

Fig. S9 shows the band alignment of the SnS_2 memtransistor before and after contact. The electron affinitiy and the energy band gap of SnS_2 were previously reported to be 4.22 eV and 2.11 eV, respectively [J. Phys, Chem. C, 122, pp. 3523-3532, 2018]. To estimate the band alignment, Fermi-level position in SnS_2 is calcuatled as follows.

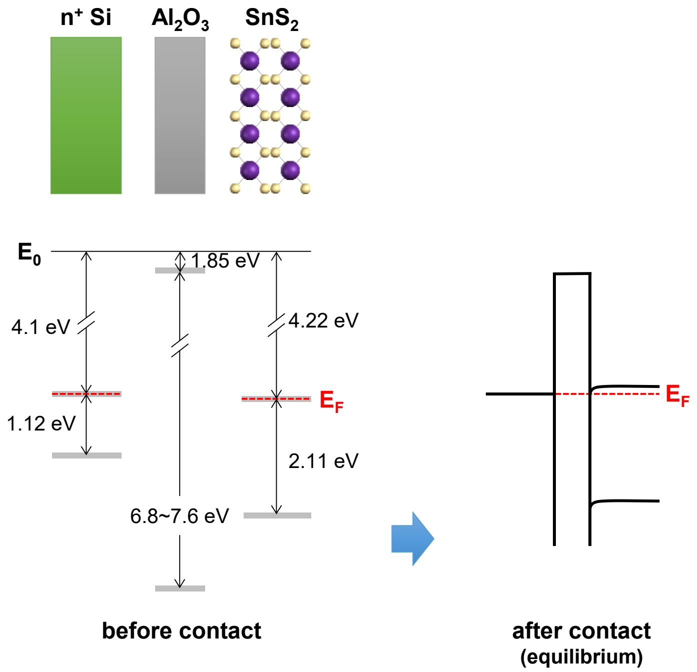

Figure S9. The energy band diagram of the SnS_2 memtransistor at equilibrium.

Step 1: Majority carrier concentration per unit area at $V_G = 0$ V is

$$n_{SnS2} = \frac{C_{Al2O3}\left|V_{th} - V_G\right|}{q} = 2.61\times10^{12}[cm^{-2}],$$

where the threshold voltage ($V_{th} = -1.35$ V) is extracted based on the extrapolation in the linear region, which finds the gate-voltage axis intercept of the linear extrapolation of the transfer curve at the first maximum of transconductance.

Step 2: The Fermi-level position can be estimated by

$$n^* = \int_{E_c}^{\infty} N(E)f(E)dE \approx N_c \exp\left(-\frac{E_c - E_F}{kT}\right),$$

where n^* is the number of electrons per unit volume, N_c is the effective density of states in the conduction band, k is the Boltzmann constant, and T is the temperature. Assuming that the channels

in the SnS_2 memtransistor was completely depleted under given gate voltages. Thereby, the number of unit volume can be simply calculated by dividing the carrier concentration by the thickness (t_{SnS2} = 20 nm), consequently $n^* = 1.3 \times 10^{18} [cm^{-3}]$. N_c is equal to $2\left(\frac{2\pi m_n^* kT}{h^2}\right)^{3/2}$, where m_n^* is the effective mass of the electron ($0.43m_0$ for SnS_2) [Sci. Rep., 7, p. 8914, 2017], giving $N_c = 7.08 \times 10^{18} [cm^{-3}]$.

Step 3: The difference between the conduction band edge and Fermi-level (E_c−E_F) is subsequently calculated to be 0.04 eV. Therefore, The Fermi-level is very closed to the conduction band edge in the SnS_2.

4-6. Reproducibility of the SnS_2 memtransistor fabrication

Fig. S10 shows the measured I_D-V_G curves from 6 devices fabricated using the same process. There is a slight device-to-device variation. Because the transfer method of the SnS_2 layer is a manual process, we believe that that the variation is owing to the thickness variation of the SnS_2 layer for each device. Nevertheless, this variation is not an issue in the operation of our analog-digital hybrid computing platform. Regardless of the device variation, the desired channel conductance can be precisely adjusted through the update-verify feedback method discussed in Supplementary Note 4-3.

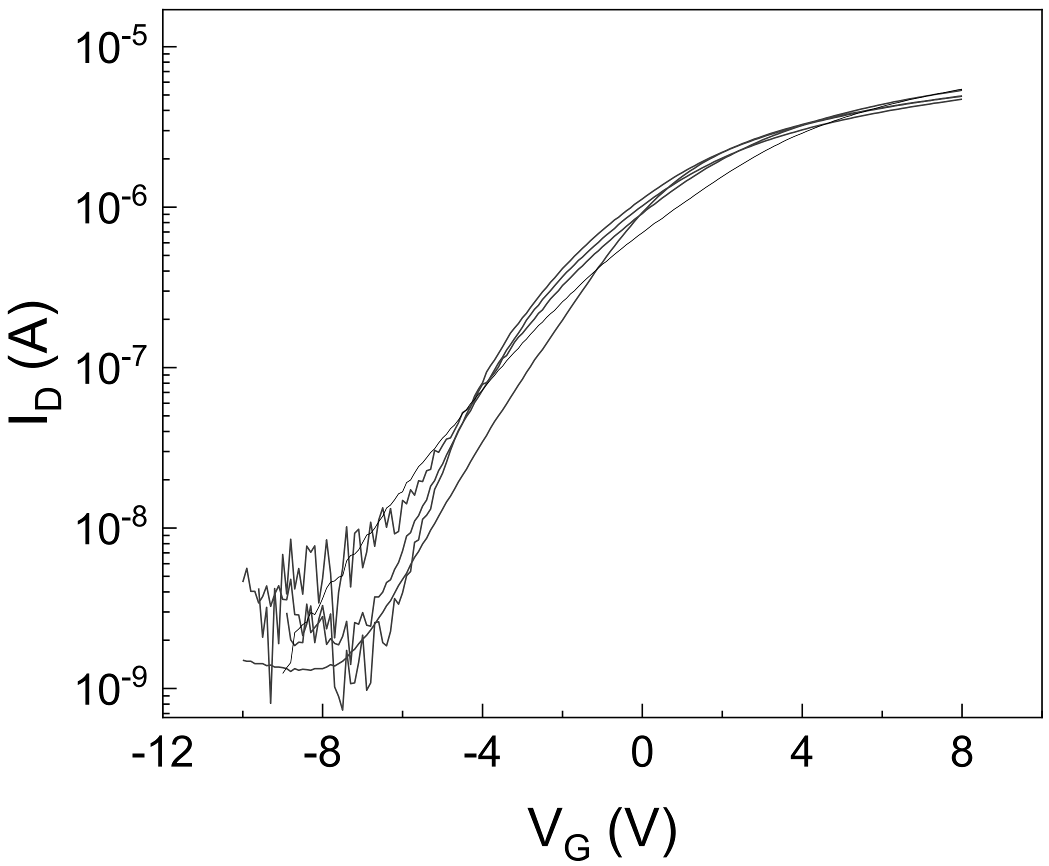

Figure S10. Measured I_D-V_G curves from 6 different devices.

Supplementary Note 5. The continuous-time Kalman filter algorithm and its analog circuit

5-1. The continuous-time Kalman filter algorithm (Derivations of Eq. (2))

From the continuous-time Kalman filter theory, the system model (Eq. (S6)) and the measurement model (Eq. (S6)) can be expressed as continuous form,

$$\begin{aligned} \dot{x}(t) &= Fx(t) + Bu(t) + Gw(t) \\ z(t) &= Hx(t) + v(t) \\ w &\sim (0,Q),\ v \sim (0,R) \end{aligned} \tag{S18}$$

Then, the update equation is given by

$$\dot{\hat{x}}(t) = F\hat{x}(t) + Bu(t) + K(t)\left[z - H\hat{x}(t)\right] \text{ where } K(t) = P(t)H^{T}R^{-1} \tag{S19}$$

It is assumed that the drone maintains a constant Euler angles within a short sampling period, so state transition matrix F can be approximated to 1 (identity matrix). In addition, the Euler angle is not affected by an external control input, so B is 0. Actually, the Kalman gain $K(t)$ is updated according to Q and R (process and measurement noises). However, because $K(t)$ is converged to a specific value as the iteration of the algorithm is repeated; thus, $K(t)$ can be regarded as a constant value K. Consequently, Eq. (S19) can be simplified as

$$\dot{\hat{x}}(t) = \dot{\hat{x}}(0) + K\left[z - \hat{x}(t)\right] \tag{S20}$$

In our study, the state vector $x(t)$ is the Euler angles of the drone ($E(t)$). Therefore,

$$\dot{E}_{est}(t) = \varpi_{est}(t) + K\left[E_{mea}(t) - E_{est}(t)\right] \tag{S21}$$

where $E_{mea}(t)$ is the Euler angles obtained by the accelerometer, and $E_{est}(t)$ is the output of the Kalman filter. Here, $\varpi_{est}(t) = \varpi_{mea}(t) - bais(t)$ where it is reasonable to assume that the bias error from the gyroscope is a constant; thus the bias error can be measured directly from the voltage output of the gyroscope. Finally, Eq. (S21) can be reconstructed as

$$\dot{E}_{est}(t) = \varpi_{mea}(t) + K\left[E_{mea}(t) - E_{est}(t)\right] \tag{S22}$$

which is Eq. (2) shown in the main text.

5-2. The analog Kalman filter circuit

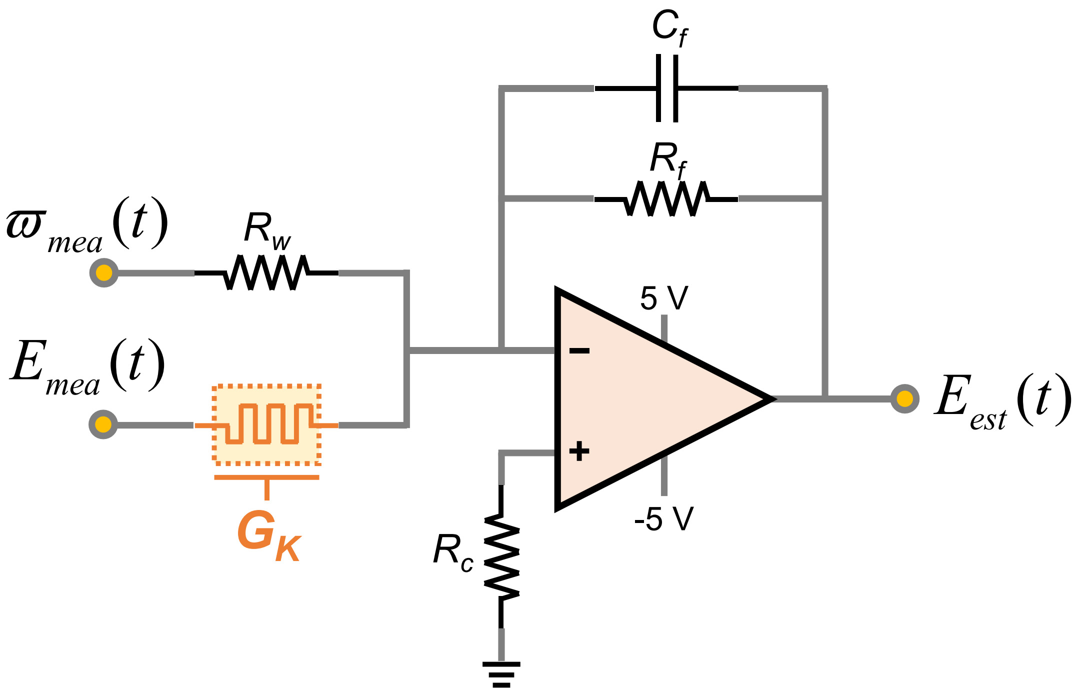

Figure S11. The analog Kalman filter circuit, which is the copy of Fig. 4b. We used an operational amplifier (Linear Technology, LT6005) for the circuit configuration. When the Kalman gain (K) is initially set, R_w and C_f are automatically determined by Eq. (S25). Because R_c does not affect the output of the circuit according to Eq. (S23), R_c was set arbitrarily as 100 kΩ. R_f was optimized through circuit simulation. Consequently, R_w = 100 kΩ, R_f = 700 kΩ, R_c = 100 kΩ, and C_f = 10 μF.

The output of the analog Kalman filter circuit, $E_{est}(t)$, is determined by

$$E_{est}(t) = -\frac{G_K}{C_f}\int E_{mea}(t)dt - \frac{1}{R_w C_f}\int w_{mea}(t)dt + E_{est}(0) \tag{S23}$$

As a differential form,

$$\dot{E}_{est}(t) = -\frac{G_K}{C_f} E_{mea}(t) - \frac{1}{R_w C_f} w_{mea}(t) \tag{S24}$$

As compared with Eq. (S24) and Eq. (S21), it can be concluded that

$$K = \frac{G_K}{C_f},\ 1 - K = \frac{1}{R_w C_f} \ \Rightarrow\ \therefore C_f - G_K = \frac{1}{R_W} \tag{S25}$$

where negative sign is eliminated by using an unity gain amplifier. As G_k increase, K and R_w are increased accordingly.

Actually, two memtransistors are required for each input signal ($E_{mea}(t)$ and $\varpi_{mea}(t)$) to fuse the signals according to the Kalman gain. However, in our experiment, one memtransistor was intentionally utilized for an easier understanding of the sensor fusion algorithm (i.e., Kalman filtering). In the Kalman filtering process, there is only one variable (i.e., Kalman gain K) that needs to be continuously updated. Therefore, we corresponded one Kalman gain value to one memtransistor conductance. Although G_K, R_w, and C_f contribute to determining the Kalman gain value in the analog Kalman filter circuit, we used a single memtransistor to effectively convey our idea that the Kalman gain can be stored in a non-volatile memtransistor.

5-3. The analog Kalman filtering result for pitch angle

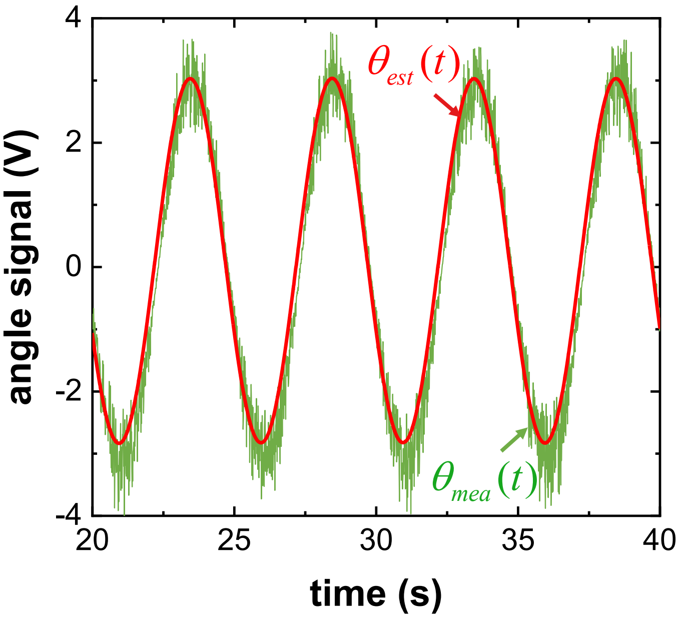

Figure S12. The noisy input signal ($\theta_{mea}(t)$) and filtered output signal ($\theta_{est}(t)$) of the analog Kalman filter circuit for the pitch angle.

5-4. The output of the analog Kalman filter with different *K*

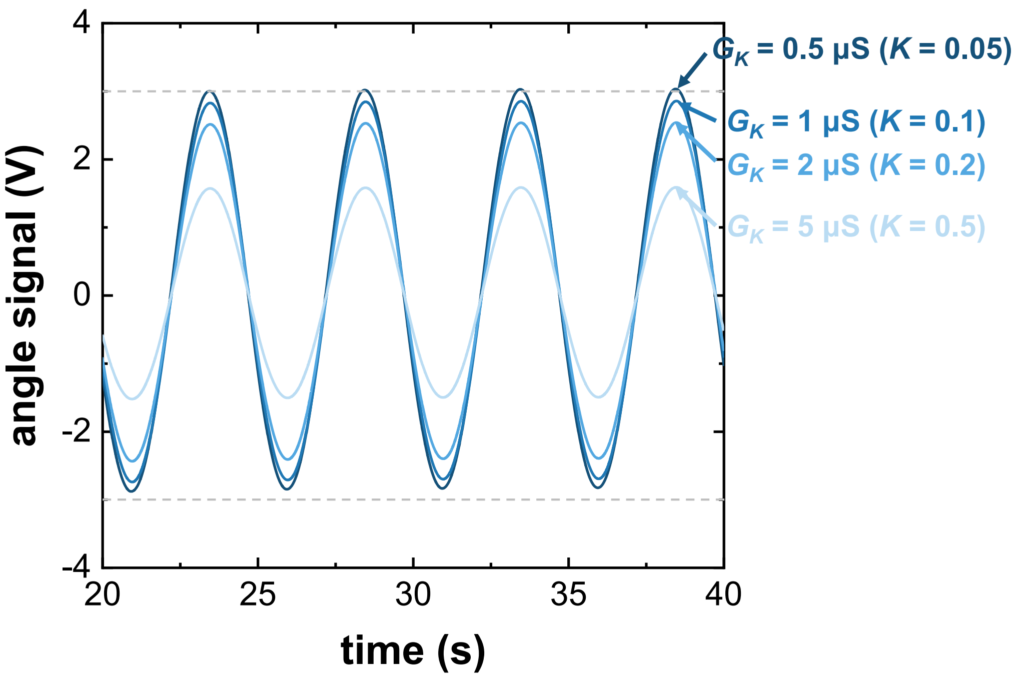

Figure S13. The output of the analog Kalman filter with different *K*.

Supplementary Note 6. The evaluation of the power consumption

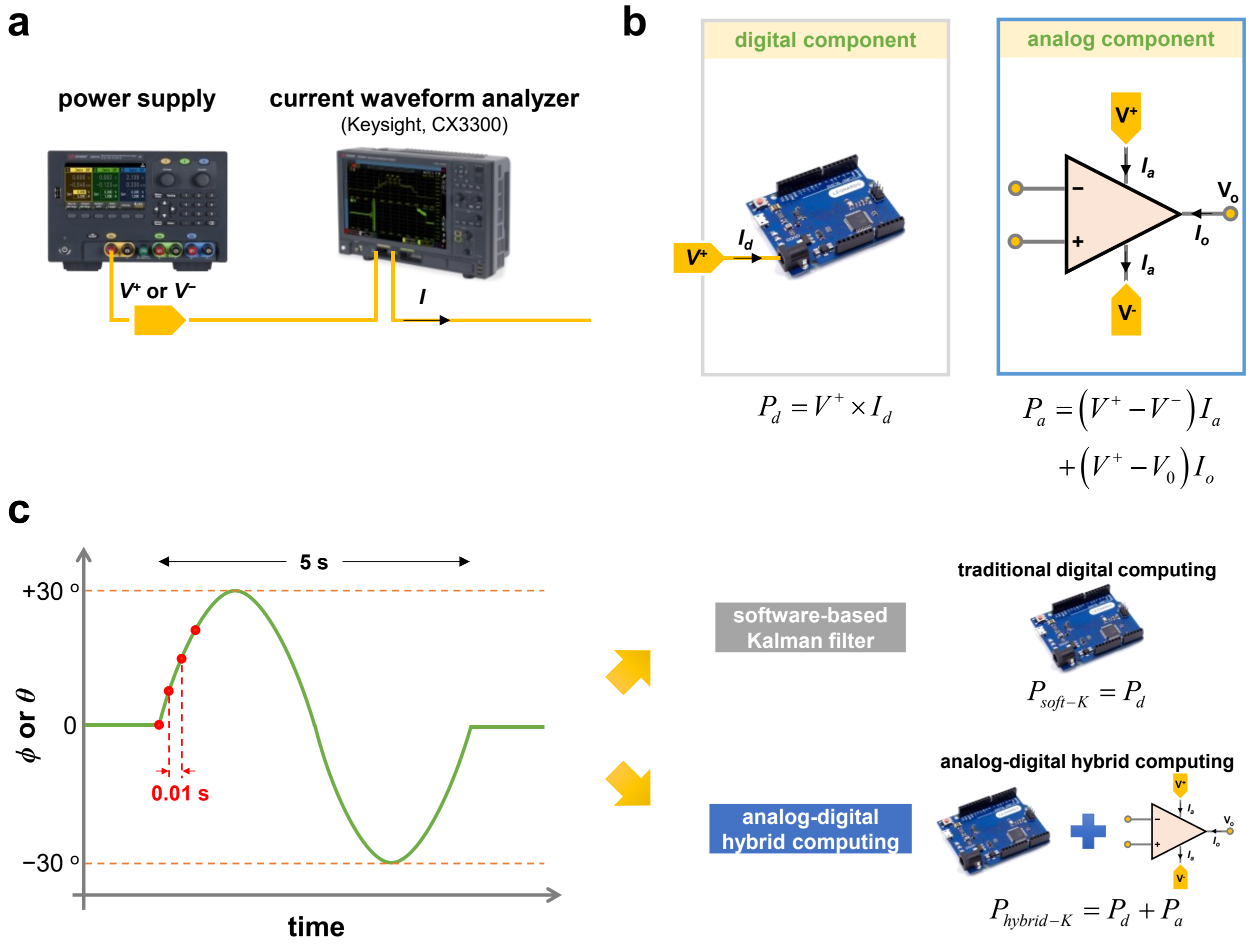

Figure S14. The comparison between the output of the analog Kalman filter circuit and the output of the software-based Kalman filter.

a software-based Kalman filter

	digital component
V^+	+5 V
$I_d(\max)$	12.3 mA
$I_d(\min)$	7.5 mA
$\int_{t=0}^{t=5s} P_{soft-K}\, dt$	197 mJ

b our hybrid computing-based Kalman filter

	digital component	analog component
V^+	5 V	+5 V
V^-		−5 V
$I_d(\max)$	5.5 mA	
$I_d(\min)$	2.2 mA	
$I_a(\max)$		+41 μA
$I_a(\min)$		+4 μA
$I_o(\max)$		+37.5 μA
$I_o(\min)$		−37.5 μA
$\int_{t=0}^{t=5s} P_d\, dt$	53.7 mJ	
$\int_{t=0}^{t=5s} P_a\, dt$		0.79 mJ
$\int_{t=0}^{t=5s} P_{hybrid-K}\, dt$	54.5 mJ	

Figure S15. The summarized results of evaluating the power consumption.

Supplementary Note 7. The comparison between the memristor and memtransistor

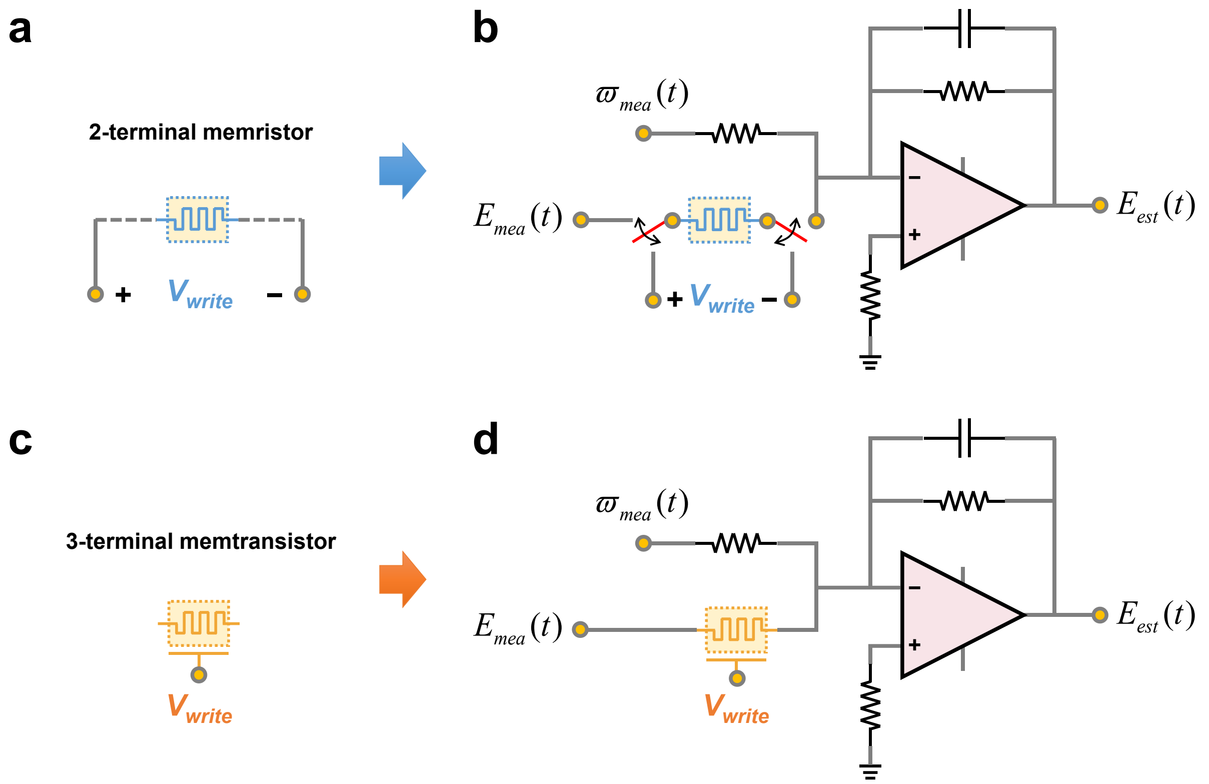

Figure S16. (a) Memristor with two electrodes. (b) Example analog circuit with a memristor. (c) Memtransistor with three electrodes. (d) Example analog circuit with a memtransistor.

A memristor has only two electrodes, thereby V_{write} (i.e., voltages for adjusting the conductance) must be applied to both ends of the two electrodes (Fig. S16a). However, these electrodes are also used for signal input/output. Consequently, additional peripheral circuits are required to distribute the input signal ($E_{mea}(t)$) and V_{write} properly (Fig. S16b).

In contrast, a memtransistor has three electrodes, and the conductance can be adjusted by using the gate electrode independently (Fig. S16c). Therefore, the conductance can be adjusted without additional peripheral circuits (Fig. S16d). In summary, there is no difference between the memristor and memtransistor in the conductance change characteristic itself, but the memtransistor enables simpler circuit configuration.
